# The prognostic role of platelet-to-lymphocyte ratio in patients with acute heart failure: A cohort study

**DOI:** 10.1038/s41598-019-47143-2

**Published:** 2019-07-23

**Authors:** Gui-lian Ye, Qiang Chen, Xueyu Chen, Ying-ying Liu, Ting-ting Yin, Qing-he Meng, Ying-chao Liu, Huai-qing Wei, Qing-hua Zhou

**Affiliations:** 1 Department of Surgery, Shandong Provincial Taishan Hospital, Taian, 271000 Shandong China; 2 School of Public Health, Shandong First Medical University and Shandong Academy of Medical Sciences, Taian, 271016 Shandong China; 3 Department of Cardiology, Taian Central Hospital, Taian, 271000 Shandong China

**Keywords:** Prognostic markers, Heart failure

## Abstract

Identification of rapid, inexpensive, and reliable prognostic factors can improve survival estimation and guide healthcare in patients with acute heart failure (AHF). In this study, we aimed to determine the prognostic value of the platelet-to-lymphocyte ratio (PLR) in patients with AHF. A total of 443 patients from two hospitals met the inclusion criteria from January 2010 to December 2017. Univariate and multivariate Cox analyses were performed to determine the association of PLR with survival. All-cause mortality was analysed using the Kaplan-Meier method. The 6-month survival rate for patients according to PLR quartiles (<110.63, 110.63–139.23, 139.23–177.17, and >177.17) were 90.09%, 76.79%, 50.07%, and 37.27%, respectively (p < 0.001). Univariate analysis identified high PLR (>110.63), old age (≥73 years), smoking habit, low estimated glomerular filtration rate (<57), and high platelet count (≥198 × 10^9^/l) as poor prognostic factors for survival. In the multivariate analysis, after adjusting for confounding factors, the third (hazard ratio [HR] = 3.118, 95% confidence interval [CI] = 1.668–5.386, p < 0.001) and fourth (HR = 2.437, 95% CI = 1.302–3.653, p < 0.001) quartiles of PLR were identified as independent prognostic factors in patients with AHF. A higher PLR was associated with poor clinical outcomes in patients with AHF and might be a novel marker in AHF management.

## Introduction

Acute heart failure (AHF), characterized by an acute deterioration in cardiac function, is a major public health problem with substantial associated economic costs and high associated risks of mortality and morbidity^1^. The prevalence of AHF was estimated to be 4.2 million in China^2^ and 23 million worldwide^3^. Despite utilization of modern pharmacological and mechanical approaches, the all-cause one-year mortality rate remains as high at 32%, and little improvement has been made in AHF outcomes^4^. Therefore, a novel factor that could aid in predicting AHF outcome is important for healthcare providers to deliver appropriate care and improve patient risk.

A simple, cheap, rapid, and reliable prognostic marker for patients with AHF is clearly needed. Inflammation and thrombosis are central pathways implicated in the generation and progression of AHF^5^. The platelet-to-lymphocyte ratio (PLR) is a novel inflammatory marker that can be applied in many diseases for predicting inflammation and mortality^6–9^. Recently, many studies indicated that PLR is a strong and independent prognostic factor in patients with cardiovascular disease^10–13^. For example, Pourafkari *et al*.^14^ found that higher PLR was associated with long-term mortality, but failed to independently predict the prognosis of AHF. Moreover, Durmus *et al*.^15^ reported that PLR was higher in patients with heart failure compared to matched controls, but this was not sufficient to establish a diagnosis of heart failure. However, whether PLR is a real risk factor or an epiphenomenon in AHF remains unclear. Furthermore, to the best of our knowledge, no study has investigated the association of PLR with mortality in AHF in China.

Thus, we conducted this cohort study to evaluate the value of PLR in predicting mortality in patients with AHF. We tested the hypothesis that higher PLR levels were associated with a higher all-cause mortality.

## Methods

**Figure 1:**
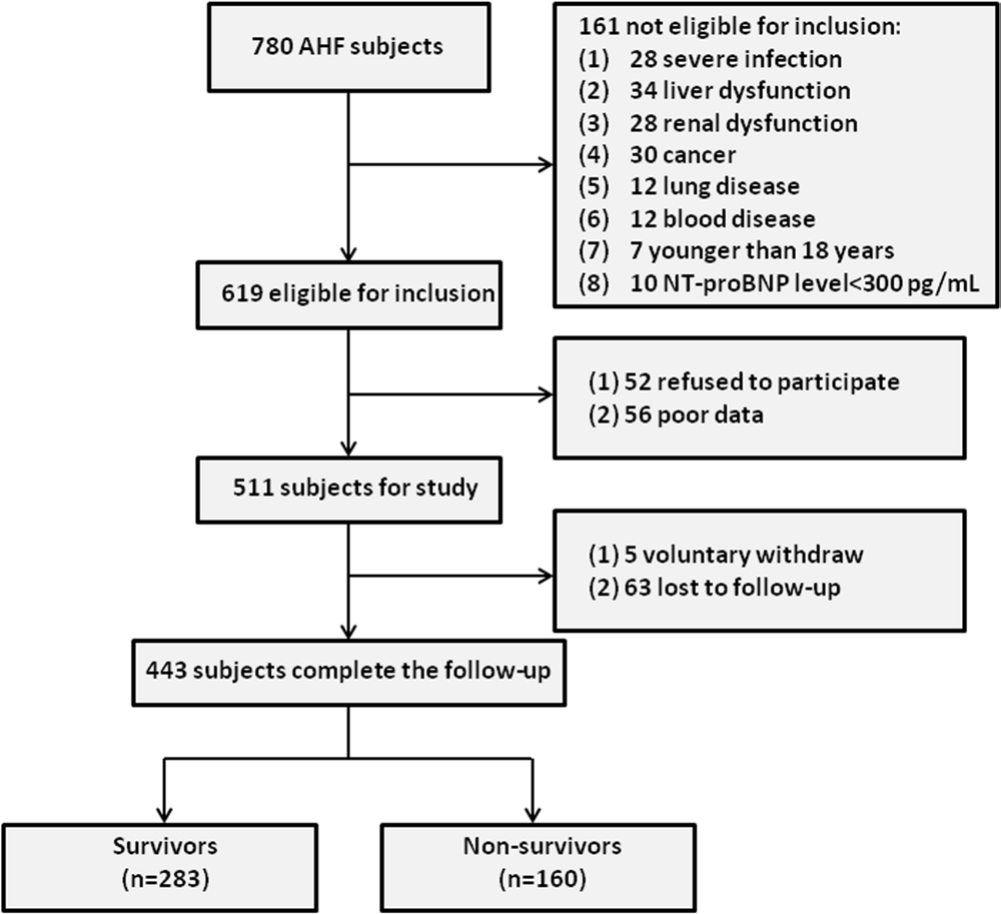
The study cohort flow diagram.

In this retrospective cohort study, patients with AHF were consecutively recruited in a Shandong Provincial Taishan Hospital and Taian Central Hospital from January 2010 to December 2017. This study was carried out according to the tenets of the Declaration of Helsinki and approved by the committee of the Shandong Provincial Taishan Hospital and Taian Central Hospital. Written consent was obtained from the patients. The diagnosis of AHF was confirmed by two cardiologists using echocardiography and electrocardiogram according to the current Chinese Society of Cardiology Heart Failure guideline^7^. Overall, 780 patients with AHF were initially enrolled, among whom 347 were later excluded. Figure 1 shows the study cohort flow diagram.

All patients underwent a standardized clinical and blood examination that consisted of physical examination, electrocardiogram, echocardiography, chest radiography, left ventricular ejection fraction (LVEF) evaluation, and testing for infection disease, liver function, and renal function. Atrial fibrillation was identified by electrocardiogram. LVEF values were obtained by performing transthoracic echocardiography performed during the admission and calculated using the modified Simpson’s rule.

The endpoint of the study included in-hospital all-cause mortality and out-of-hospital all-cause mortality. Information regarding death was obtained from medical records. All the discharge patients with AHF regularly visited the clinic (typically every month) if the physical condition of the patient permitted. If not, patients with AHF were followed up over the telephone, and outpatient records were obtained every month to remain up-to-date on patient survival status, disease progress, and time of death. All patients with AHF were followed up for 6 months after admission; the last follow-up was June 30, 2018.

Clinical and demographic information was obtained from the medical data platform by two independent investigators. This included the following demographic information: age, sex, red blood cell (RBC) count, white blood cell (WBC) count, neutrophil count, lymphocyte count, systolic blood pressure (SBP), diastolic blood pressure (DBP), body mass index (BMI), creatinine, platelet count, urea nitrogen, total protein, hypertension, diabetes, smoking, drinking, estimated glomerular filtration rate (eGFR), NT-proBNP, heart rate, diuretic use, LVEF, use of aldosterone antagonists, angiotensin converting enzyme inhibitors/angiotensin receptor blocker (ACEI/ARB), or beta-blockers, atrial fibrillation, valvular heart disease, cardiomyopathy, and coronary heart disease.

Venous blood samples were drawn immediately after hospital admission. Blood samples were collected from each patient using ethylenediaminetetraacetic acid tubes to prevent blood coagulation. Two ml of blood was used to perform a complete blood count - including the RBC, WBC, neutrophil, platelet, and lymphocyte counts - for each patient using the Sysmex automatic blood counting system (Tokyo, Japan). Laboratory analysis was performed using a commercially available kit (Roche Diagnostics GmbH, Mannheim, Germany).

All statistical analyses were performed using SPSS 13.0 software (IBM-SPSS, Chicago, IL, USA). Continuous variables are presented as mean ± standard deviation. The Chi-square test and independent Student’s t-test were used for comparisons of categorical clinical characteristics between survivors and non-survivors. The one-way analysis of variance and Chi-square test were used for comparing the participants’ characteristics among the four groups divided according to PLR quartile. The variables were dichotomized based on the mean value of each variable. Pearson analysis was performed to assess the relationship of platelet and lymphocyte counts with PLR. To identify independent predictors of mortality for the cohort, univariable and multivariable Cox proportional hazards models were used. The patient clinical end points were calculated using the Kaplan-Meier method and compared using the log-rank test. A two-sided p value < 0.05 was considered statistically significant.

### Ethical approval

This study was approved by the ethical committee of the Shandong Provincial Taishan Hospital and Taian Central Hospital.

### Informed consent

Written informed consent for the use of any clinical data in research was obtained for all patients.

## Results

Baseline characteristics of the survivor and non-survivor groups are presented in Table 1. Overall, 149 male and 294 female patients with AHF were included in the study cohort. During a mean longitudinal follow-up time of 143.68 days (range, 20–180 days), there were 160 deaths (36.12%). In this study, a total of 160 events were recorded during the 6-month follow-up period, with 58 deaths during in-hospital care, 92 deaths post discharge, 66 due to HF, 29 due to sudden cardiac death, 27 due to other cardiovascular causes, 33 due to non-cardiovascular causes (including renal failure, cancer, respiratory failure, cerebrovascular disease), and 5 classified as unknown. Among the survivors, the mean age was 72.34 ± 6.80 years; for non-survivors, the mean age was 71.61 ± 6.84 years (t = 1.083, p = 0.280). There was no significant difference in the age, sex, WBC count, RBC count, neutrophil count, DBP, BMI, creatinine, urea nitrogen, total protein, history of hypertension, diabetes, smoking, or drinking, eGFR, heart rate, use of diuretics, aldosterone antagonists, ACEI/ARB, or beta-blockers, atrial fibrillation, valvular heart disease, cardiomyopathy, or coronary heart disease between the survivor group and non-survivor groups (p > 0.05). The platelet level was significantly higher (p < 0.001) in the non-survivor group (233.54 ± 52.01) than in the survivor group (189.86 ± 49.23). The PLR was significantly higher (p < 0.001) in the non-survivor group (173.81 ± 54.51) than in the survivor group (136.02 ± 45.89).

**Table 1:** Baseline demographic and lifestyle characteristics in patients with acute feart failure.

	Survivors (n = 283)	Non-survivors (n = 160)	t value	P value
Age (years)	72.34 ± 6.80	71.61 ± 6.84	1.083	0.280
Male/Female	100/183	49/111	1.016	0.313
White blood cell (10^9^/l)	6.01 ± 1.81	6.15 ± 1.84	0.797	0.426
Red blood cell (10^12^/l)	4.26 ± 0.42	4.32 ± 0.42	1.529	0.127
Platelet (10^9^/l)	189.86 ± 49.23	233.54 ± 52.01	8.788	<0.001
Neutrophil (10^9^/l)	4.01 ± 1.45	4.19 ± 1.67	1.155	0.249
Lymphocyte (10^9^/l)	1.49 ± 0.48	1.42 ± 0.38	1.719	0.086
SBP (mm Hg)	76.34 ± 8.86	75.34 ± 9.34	1.125	0.261
DBP (mm Hg)	133.57 ± 15.47	130.63 ± 12.97	2.143	0.033
BMI (Kg/m^2^)	22.65 ± 3.43	22.09 ± 3.10	1.274	0.204
NT-proBNP (pg/ml)	5138.25 ± 550.24	5264.36 ± 882.67	1.851	0.065
Creatinine (umol/l)	71.31 ± 16.70	69.83 ± 16.18	1.132	0.183
Urea nitrogen(mmol/l)	6.05 ± 1.67	5.93 ± 1.68	0.731	0.465
Total protein (g/l)	72.40 ± 5.24	72.49 ± 5.21	0.188	0.851
PLR	136.02 ± 45.89	173.81 ± 54.51	7.770	<0.001
Hypertension (yes/no)	98/185	49/110	0.666	0.414
Diabetes(yes/no)	28/255	10/150	1.731	0.188
Smoking (yes/no)	61/222	33/127	0.053	0.818
Drinking (yes/no)	115/168	62/98	0.152	0.697
Left ventricular ejection fraction	45.52 ± 9.74	43.58 ± 9.09	2.064	0.040
eGFR (ml/min/1.73 m^2^)	57.96 ± 13.07	57.93 ± 12.87	0.026	0.979
Heart rate	83.93 ± 14.15	84.18 ± 14.07	0.176	0.860
Diuretics (yes/no)	271/12	152/8	0.137	0.711
Aldosterone antagonists (yes/no)	237/46	137/23	0.275	0.600
ACEI/ARB (yes/no)	145/133	76/89	2.461	0.117
Beta-blockers (yes/no)	195/88	109/51	0.029	0.865
Atrial fibrillation (yes/no)	109/174	58/102	0.223	0.636
Valvular heart disease (yes/no)	25/258	17/143	0.382	0.563
Cardiomyopathy (yes/no)	76/207	34/126	1.720	0.190
Coronary heart disease (yes/no)	243/40	130/30	1.637	0.201

BMI: body mass index, SBP: systolic blood pressure, DBP: diastolic blood pressure, ACEI: angiotensin converting enzyme inhibitors, ARB: angiotensin receptor blocker, PLR: platelet-to-lymphocyte ratio, eGFR: estimated glomerular filtration rate.

**Figure 2:**
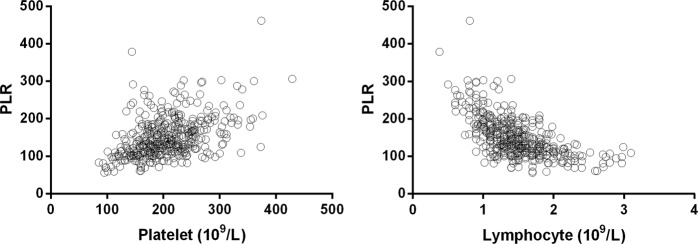
The association between platelet, and lymphocyte counts with platelet-to-lymphocyte ratio (PLR) levels of patients with acute heart failure.

In an attempt to determine whether PLR levels influence the clinical outcome of patients with AHF, we first subdivided the study patients into four groups based on PLR quartiles (<110.63 [n = 111], 110.63–139.23 [n = 112], 139.23–177.17 [n = 110], >177.17 [n = 110]) (Table 2). Higher PLR levels significantly correlated with a high rate of death (9.91%, 23.21%, 49.09%, 62.73%), high platelet count (172.92 ± 48.12, 198.38 ± 40.91, 211.46 ± 42.11, 240.20 ± 61.71), and low lymphocyte count (1.83 ± 0.48, 1.58 ± 0.33, 1.34 ± 0.27, 1.12 ± 0.32) (p for both <0.001). Furthermore, high PLR levels significantly correlated with smoking, age, and eGFR (p for both, <0.05). There was no correlation between PLR and the remaining factors (p for all, >0.05). Pearson analysis showed that there was a significant positive correction between PLR and platelet count (r = 0.455, p < 0.001), and a negative correction between PLR and lymphocyte count (r = −0.611, p < 0.001) (Fig. 2).

**Table 2:** The relation between clinico-pathological parameters and PLR levels of patients with acute heart failure.

	PLR Quartiles				
	<110.63 (n = 111)	110.63–139.23 (n = 112)	139.23–177.17 (n = 110)	>177.17 (n = 110)	F /P value for trend
Age (years)	71.25 ± 7.03	72.23 ± 6.29	73.25 ± 6.92	73.54 ± 6.92	0.045
Male/Female	37/74	40/72	36/74	36/74	0.811
Death Events	11	26	54	69	<0.001
Platelet (10^9^/l)	172.92 ± 48.12	198.38 ± 40.91	211.46 ± 42.11	240.20 ± 61.71	<0.001
Lymphocyte (10^9^/l)	1.83 ± 0.48	1.58 ± 0.33	1.34±0.27	1.12±0.32	<0.001
PLR	95.05±13.59	125.86±7.34	157.94±10.52	220.76±44.51	<0.001
SBP (mm Hg)	75.89±9.25	76.30±9.31	75.69±8.61	76.04±9.07	0.965
DBP (mm Hg)	134.32±15.29	131.57±16.20	132.16±13.43	131.99±13.58	0.507
BMI (Kg/m^2^)	22.74±3.68	22.36±3.52	22.72±3.34	22.02±2.72	0.587
Hypertension	38	36	38	35	0.864
Diabetes	11	10	9	8	0.505
Smoking	17	17	31	29	0.032
Drinking	39	49	43	46	0.628
Left ventricular ejection fraction	45.29±10.01	44.85±9.57	45.11±9.50	44.04±9.17	0.776
eGFR (ml/min/1.73 m^2^)	59.81±12.20	58.82±14.70	57.55±13.15	56.63±11.75	0.001
Heart rate	83.34±13.70	83.96±14.53	84.13±14.82	84.65±13.49	0.922
Diuretics	108	106	102	107	0.972
Aldosterone antagonists	89	95	93	97	0.655
ACEI/ARB	59	59	52	51	0.483
Beta-blockers	80	73	76	75	0.866
Atrial fibrillation	47	44	40	36	0.300
Valvular heart disease	10	7	11	14	0.264
Cardiomyopathy	24	23	33	30	0.264
Coronary heart disease	92	97	94	90	0.935

BMI: body mass index, SBP: systolic blood pressure, DBP: diastolic blood pressure, ACEI: angiotensin converting enzyme inhibitors, ARB: angiotensin receptor blocker, PLR: platelet-to-lymphocyte ratio, eGFR: estimated glomerular filtration rate.

To investigate whether PLR level and other clinicopathological factors were associated with clinical outcome of patients with AHF, univariate Cox proportional models for survival of patients with AHF were calculated (Table 3). Univariate analysis identified high PLR, old age, smoking habit, low eGFR, and high platelet count as poor prognostic factors for survival in this study cohort.

**Table 3:** Univariate and Multivariate cox proportional analysis regarding survival in patients with acute heart failure.

	Univariate analysis			Multivariate analysis		
	HR	95%CI	P value	HR	95%CI	P value
Age (years)						
<73	1			1		
≥73	1.089	1.002–1.054	0.011	0.989	0.956–1.023	0.512
Gender						
Male	1			1		
Female	1.219	0.871–1.707	0.248	1.174	0.617–2.233	0.625
PLR						
<110.63	1			1		
110.63–139.23	2.528	1.249–5.116	0.010	3.660	0.469–16.366	0.128
139.23–177.17	2.579	1.863–3.579	<0.001	3.118	1.668–5.386	<0.001
>177.17	2.126	1.718–2.631	<0.001	2.437	1.302–3.653	<0.001
Platelet						
<198	1			1		
≥198	5.171	3.522–7.593	<0.001	1.005	0.993–1.018	0.421
Lymphocyte						
<1.41	1			1		
≥1.41	0.837	0.614–1.412	0.262	1.115	0.181–6.884	0.906
NT-proBNP						
<5206	1			1		
≥5206	1.161	0.356–2.362	0.376	1.011	0.284–4.322	0.806
BMI						
<22.20	1			1		
≥22.20	0.835	0.545–1.279	0.407	0.957	0.874–1.048	0.348
Hypertension						
No	1			1		
Yes	1.009	0.790–1.289	0.942	1.111	0.525–1.580	0.740
Diabetes						
No	1			1		
Yes	0.962	0.651–1.421	0.846	1.198	0.213–1.676	0.328
Smoking						
No	1			1		
Yes	1.040	1.003–1.058	0.017	1.134	0.564–2.282	0.724
Drinking						
No	1			1		
Yes	0.945	0.745–1.198	0.638	1.169	0.054–1.532	0.324
SBP						
No	1			1		
Yes	1.111	0.979–1.000	0.053	1.001	0.994–1.006	0.323
Left ventricular ejection fraction						
<45	1			1		
≥45	0.873	0.640–1.191	0.392	0.983	0.960–1.006	0.147
eGFR						
<57	1			1		
≥57	0.927	0.908–0.990	0.001	1.022	0.994–1.052	0.125
Heart rate						
<84	1			1		
≥84	0.974	0.713–1.330	0.869	0.977	0.950–1.006	0.121
Diuretics						
No	1			1		
Yes	0.947	0.531–1.688	0.853	0.801	0.394–1.632	0.542
Aldosterone antagonists						
No	1			1		
Yes	1.042	0.760–1.428	0.800	0.618	0.209–1.826	0.383
ACEI/ARB						
No	1			1		
Yes	0.983	0.778–1.242	0.886	0.772	0.360–1.655	0.506
Beta-blockers						
No	1			1		
Yes	0.985	0.766–1.267	0.906	0.450	0.207–1.098	0.075
Atrial fibrillation						
No	1			1		
Yes	1.002	1.789–1.274	0.984	0.664	0.269–1.539	0.322
Valvular heart disease						
No	1			1		
Yes	1.102	0.731–1.662	0.642	1.166	0.541–2.514	0.695
Cardiomyopathy						
No	1			1		
Yes	1.085	0.834–1.411	0.543	0.516	0.225–1.183	0.118
Coronary heart disease						
No	1			1		
Yes	1.044	0.747–1.459	0.802	1.040	0.389–2.779	0.938

BMI: body mass index, SBP: systolic blood pressure, ACEI: angiotensin converting enzyme inhibitors, ARB: angiotensin receptor blocker, eGFR: estimated glomerular filtration rate.

After univariate Cox analysis, to investigate whether PLR level and platelet level were associated with clinical outcome of patients with AHF, a multivariate analysis using a Cox proportional hazard model was performed (Table 3). In the multivariate analysis that included age, sex, WBC count, RBC count, neutrophil count, SBP, DBP, BMI, creatinine, PLR, platelet count, urea nitrogen, total protein, history of hypertension, diabetes, smoking, or drinking, eGFR, heart rate, diuretic use, LVEF, use of aldosterone antagonists, ACEI/ARB, or beta-blockers, atrial fibrillation, valvular heart disease, cardiomyopathy, coronary heart disease, and PLR level within the third (hazard ratio [HR] = 3.118, 95% confidence interval [CI] = 1.668–5.386, p < 0.001) and fourth (HR = 2.437, 95% CI = 1.302–3.653, p < 0.001) quartile as independent prognostic factors for clinical outcome of patients with AHF.

**Figure 3:**
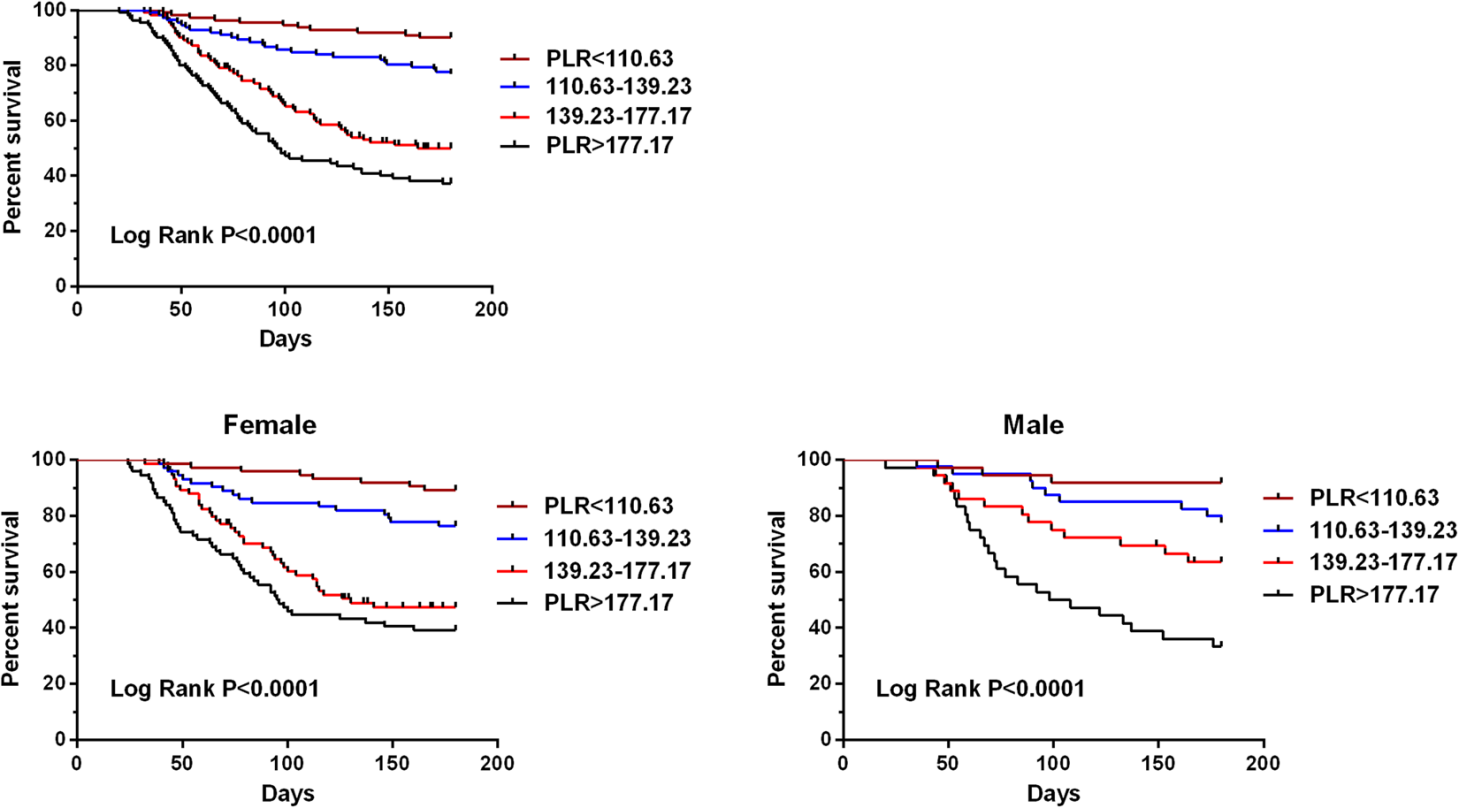
Kaplan–Meier curve stratified by platelet-to-lymphocyte ratio according to quartiles regarding all-cause survival for patients with acute heart failure.

Kaplan-Meier curves for clinical outcome of patients with AHF grouped according to quartiles of PLR are shown in Fig. 3. The pairwise log-rank test indicated significant differences between the highest quartile (PLR > 177.17) compared with the lowest (PLR < 110.63), second, (139.23 ≥ PLR ≥ 110.63), and third (177.17 ≥ PLR > 139.23) quartiles (p < 0.001). The 180 days survival rate for patients in the PLR quartiles were 90.09%, 76.79%, 50.07%, and 37.27% for the lowest, second, third, and highest quartiles, respectively (p < 0.001, Fig. 3). Similar results were also identified in the subgroups separated according to sex (p < 0.001, Fig. 3).

## Discussion

In this study, we reviewed the prognostic significance of PLR compared with other clinical factors in patients with AHF. We demonstrated that patients in the highest and third PLR quartiles had a significantly higher rate of death compared with the lowest 2 quartiles Furthermore, univariate analysis identified high PLR as a poor prognostic factor for survival in this study cohort; similar results were also found in multivariate Cox analysis after adjustment for multiple potential confounders.

Previous studies have reported that higher PLR is associated with poor clinical outcomes in various cardiovascular diseases^16–18^. PLR, a simple, inexpensive, and rapid marker that is routinely reported using automated laboratory equipment, is used to perform complete blood counts, reflects inflammation, atherosclerosis, and platelet activation^9^. Kurtul *et al*.^19^ reported that PLR at admission is significantly associated with the severity and complexity of coronary atherosclerosis in patients with acute coronary syndromes. Thomas *et al*.^20^ evaluated 2121 patients with peripheral arterial occlusive disease from 2005 to 2010 and suggested that increased PLR is significantly associated with patients at high risk for cardiovascular endpoints. In addition, Akboga *et al*.^21^ showed that PLR was found to be an independent predictor of the presence of severe coronary artery disease (odds ratio: 1.043, 95%CI = 1.036–1.049, p < 0.001). In our study, we showed that high PLR is a significant and independent predictor of long-term mortality in patients with AHF. To the best of our knowledge, limited data are available in the literature regarding the association of PLR with mortality in AHF in China.

Only five studies have examined the association of PLR with AHF, including one study of a Portuguese population and one study of a US population. For Portuguese patients, Durmus *et al*.^15^ reported that the PLR of patients with heart failure was significantly higher compared to controls, but PLR was not an independent predictor of mortality. Pourafkari *et al*.^14^ reported, in 354 patients, that higher PLR was associated with long-term mortality, but failed to independently predict the prognosis of AHF. The previously reported data are conflicting. In our study, we validated the prognostic impact of PLR level on all-cause mortality and clearly demonstrated that PLR level was independently associated with all-cause mortality in patients with AHF, based on a large cohort of 443 patients with AHF. However, as we have seen that the reported results are conflicting. Given the nature of a registry study, selection bias of different studies may be one reason leading to discordant results. Different studies also included different variables into the multivariable model, which may be another reason for the discordant results. However, although the present results are discordant, almost all the studies suggested that a higher PLR was associated with long-term mortality.

The mechanisms of the association between PLR and AHF are unclear. One possible mechanism may be an increased inflammatory response. The inflammatory response plays an important role in the pathogenesis of AHF^22,23^. Elevated C-reactive protein level is associated with adverse outcomes in patients with heart failure^24^. In addition, inflammatory cytokines may contribute to the clinical outcome of patients with AHF^22^. Recent research suggested that platelets interact with leukocytes and endothelial cells and release inflammatory factors, leading to adhesion and transmigration of monocytes; thus, platelets represent an important link between inflammation, thrombosis, and atherogenesis^25,26^. Moreover, a decreased lymphocyte count is also significantly associated with increased mortality^27^. The combination of these parameters - an elevated platelet count and decreased lymphocyte count, which lead to an elevated PLR - might therefore predict major adverse outcomes in AFH.

Physiologic stress leading to a lower lymphocyte count is another likely mechanism. Thomson *et al*.^28^ reported that cortisol and catecholamine lead to redistribution of lymphocytes to lymphatic organs, and are released in response to physiologic stress during AHF. Therefore, a high level of physiologic stress in patients with AHF might indicate high levels of cortisol and catecholamine, which can translate into a lower lymphocyte count^19^. Another underlying mechanism may be that PLR is associated with slow coronary blood flow rate^29^. Patients with AHF have a slow coronary blood flow rate^30^. In addition, a high platelet count may represent a prothrombotic state, leading to worse outcomes. Elevated PLR may indicate high physiologic stress, which contributes to poor prognosis.

There are several limitations of our study. First, our study was retrospective in design with data from a single centre, which might limit the generalizability of the results. Additionally, PLR is a marker of systematic inflammation. Although acute infectious diseases and systemic diseases were excluded, chronic subclinical inflammation in patients with AHF is still difficult to detect through medical screenings.

## Conclusion

In conclusion, high PLR level was a significant and independent prognostic factor associated with clinical outcome in patients with AHF. As a simple, inexpensive, and rapid parameter, PLR can be useful for predicting prognosis of patients with AHF. Further studies are needed to investigate the mechanism of this correlation and confirm its role in the disease process.

## Data Availability

All data generated or analysed during this study are included in this published article.
